# Coronary Perforation During Excimer Laser Angioplasty in a Patient With Prior Thoracic Radiotherapy

**DOI:** 10.1002/ccd.70792

**Published:** 2026-07-30

**Authors:** Francesco Maria Sparasci, Alessandro Cafaro, Dionigi Fischetti, Alessandro Mandurino‐Mirizzi

**Affiliations:** ^1^ Department of Molecular Medicine University of Pavia Pavia Italy; ^2^ Division of Cardiology, “Vito Fazzi” Hospital Lecce Italy; ^3^ Department of Experimental Medicine (DiMeS) University of Salento Lecce Italy

**Keywords:** calcified coronary lesions, complex percutaneous coronary intervention, plaque modification, vascular fragility

## Abstract

**Background:**

Management of heavily calcified coronary lesions remains challenging despite advances in plaque‐modifying technologies. Excimer laser coronary angioplasty (ELCA) is an effective adjunctive strategy with a low risk of no‐reflow.

**Case Summary:**

A 75‐year‐old woman with non–ST‐segment elevation acute coronary syndrome and severe left ventricular dysfunction, previously treated with thoracic radiotherapy, underwent percutaneous coronary intervention of a calcified left anterior descending artery lesion. Optical coherence tomography revealed an eruptive calcified nodule with thrombus and a concentric stenosis. ELCA was selected to minimize distal embolization; however, the procedure was complicated by multiple coronary perforations requiring covered stent implantation.

**Discussion:**

This case suggests that coronary perforation during ELCA may result from the interplay between lesion complexity, procedural factors, and radiation‐related vessel wall remodeling.

## Take‐Home Messages

- ELCA is effective for complex lesions but may increase procedural risk in irradiated vessels.
- Careful device selection and technique are essential in this population.

**Abbreviations**
ACS: Acute coronary syndrome
CICU: Coronary intensive care unit
ELCA: Excimer laser coronary angioplasty
LAD: Left anterior descending (artery)
LV: Left ventricle/left ventricular
LVEF: Left ventricular ejection fraction
NSTE‐ACS: Non–ST‐segment elevation acute coronary syndrome
OCT: Optical coherence tomography
PCI: Percutaneous coronary intervention
TIMI: Thrombolysis In Myocardial Infarction

## 1 History of Presentation

A 75‐year‐old woman presented to the Emergency Department on January 20, 2026, with progressive dyspnea and fatigue over several days. She reported no angina at rest.

On examination, heart sounds were regular without murmurs. Breath sounds were reduced at both lung bases with diffuse fine crackles. The remainder of the examination was unremarkable. Vital signs: blood pressure 145/75 mmHg, heart rate 75 bpm, oxygen saturation 96% on room air, respiratory rate 26 breaths/min.

## 2 Past Medical History

Cardiovascular risk factors included type 2 diabetes mellitus, hypertension, former smoking (≈10 pack‐years), overweight, dyslipidemia, and noncritical supra‐aortic atherosclerosis. Other comorbidities were hypothyroidism, left‐sided breast cancer treated with quadrantectomy, chemotherapy, and radiotherapy (2024), and colonic diverticulosis.

Prior cardiovascular history was unremarkable. At the last cardiology evaluation (June 2025) for occasional exertional angina, echocardiography showed normal biventricular function without significant valvular disease. Home medications were acetylsalicylic acid 100 mg once daily, linagliptin 5 mg once daily, olmesartan/amlodipine 40/5 mg once daily, nebivolol 5 mg once daily, esomeprazole 20 mg once daily, levothyroxine (100 mg 4 days/week, 50 mg 3 days/week), and atorvastatin 20 mg once daily.

## 3 Differential Diagnosis

The leading diagnosis was acute coronary syndrome with early congestive heart failure. Differential diagnoses included acute respiratory distress syndrome and anemia.

## 4 Investigations

Electrocardiogram showed sinus rhythm (78 bpm), normal atrioventricular conduction, left anterior fascicular block, no ischemic changes, and one premature ventricular complex (Figure 1). Laboratory tests: hemoglobin 12.5 g/dL, platelets 170,000/µL, white blood cells 7430/µL. High‐sensitivity troponin I was elevated (0–1 h points 1254–1990 ng/L; reference < 54 ng/L). C‐reactive protein was normal (2.0 mg/L). Renal and hepatic function were within normal limits. Chest X‐ray showed interstitial edema without consolidations or pleural effusion. Transthoracic echocardiography demonstrated severe left ventricular systolic dysfunction with anterior and anteroseptal hypokinesia and a left ventricular ejection fraction (LVEF) of 35%, mild mitral regurgitation, and no significant aortic valve disease.

**Figure 1:**
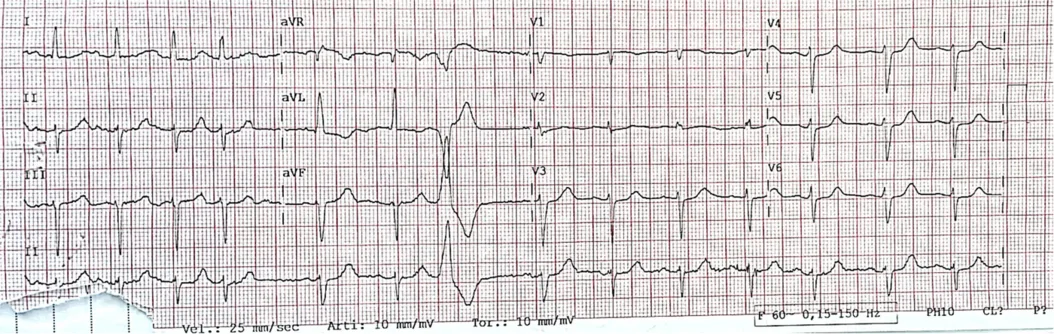
Baseline 12‐lead electrocardiogram showing sinus rhythm at 78 bpm, normal atrioventricular conduction, left anterior fascicular block, no ischemic changes, and one premature ventricular complex. [Color figure can be viewed at wileyonlinelibrary.com]

## 5 Management (Medical/Interventions)

The presentation was consistent with non–ST‐segment elevation acute coronary syndrome (NSTE‐ACS) complicated by an acute heart failure with reduced ejection fraction, and the patient was admitted to the coronary intensive care unit (CICU). Intravenous furosemide (20 mg twice daily) and bisoprolol (3.75 mg once daily) were initiated.

Coronary angiography (January 21, 2026; right radial access) showed critical tandem lesions in the proximal left anterior descending artery (LAD): a first eccentric calcified lesion followed by a concentric calcified stenosis (Figure 2). Given clinical stability, TIMI flow grade ≥2, and the anatomical complexity related to heavily calcified lesions, PCI was deferred. After exclusion of surgery due to comorbidities (including prior left chest radiotherapy), dual antiplatelet therapy (aspirin and ticagrelor) was started, and deferred PCI was planned after heart failure stabilization.

**Figure 2:**
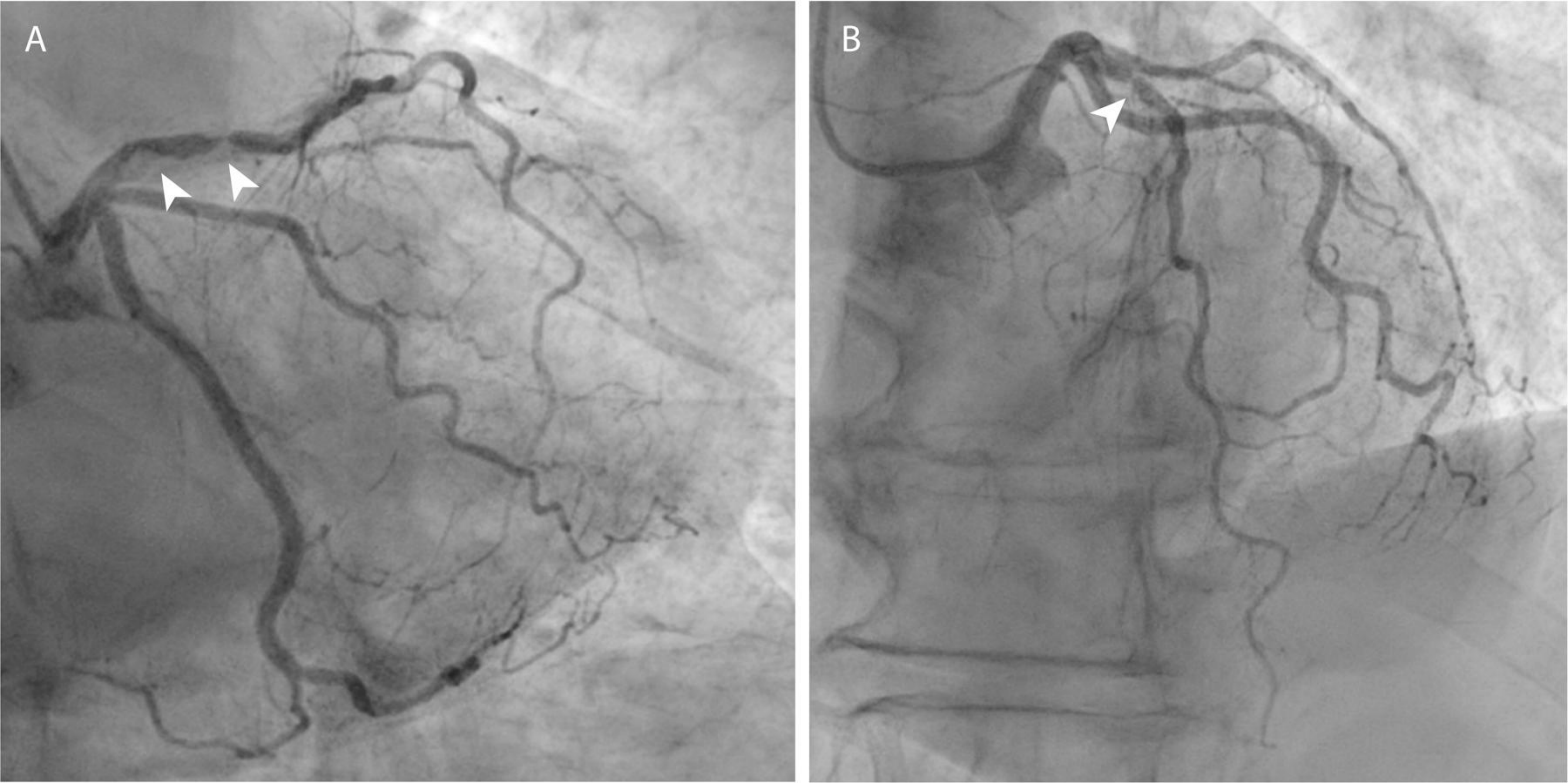
Coronary angiography of the left coronary artery showing a heavily calcified left anterior descending artery with two critical proximal stenoses. (A) Caudal projection demonstrating both lesions (arrowheads). (B) Cranial projection demonstrating the more distal concentric stenosis (arrowhead).

PCI was performed on January 27, 2026, via left radial access using a 7‐F EBU 4.0 guiding catheter (detailed equipment list in Figure 3). After guidewire crossing and predilatation with a 2.0‐mm noncompliant balloon, optical coherence tomography (OCT) revealed long lesions characterized by an eruptive calcified nodule, followed by a concentric calcified stenosis, with superficial thrombus (Figure 4). In addition, OCT of adjacent non‐atherosclerotic segments showed diffuse intimal thickening with medial thinning (Figure 5). No significant fractures of the calcified plaques were observed after predilatation, and a suboptimal result was anticipated without dedicated calcium modification. Excimer laser coronary angioplasty (ELCA) was performed using a 0.9 mm catheter with multiple antegrade and retrograde runs (80 mJ/mm^2^, 80 Hz) up to the mid‐LAD, achieving adequate lesion crossing (Video S1). Laser energy was delivered in an on–off sequence, consisting of 10 s of activation followed by 5 s of rest, for a total irradiation time of approximately 5 min. Continuous saline flush was used during laser delivery, and no contrast medium was injected during activation.

**Figure 3:**
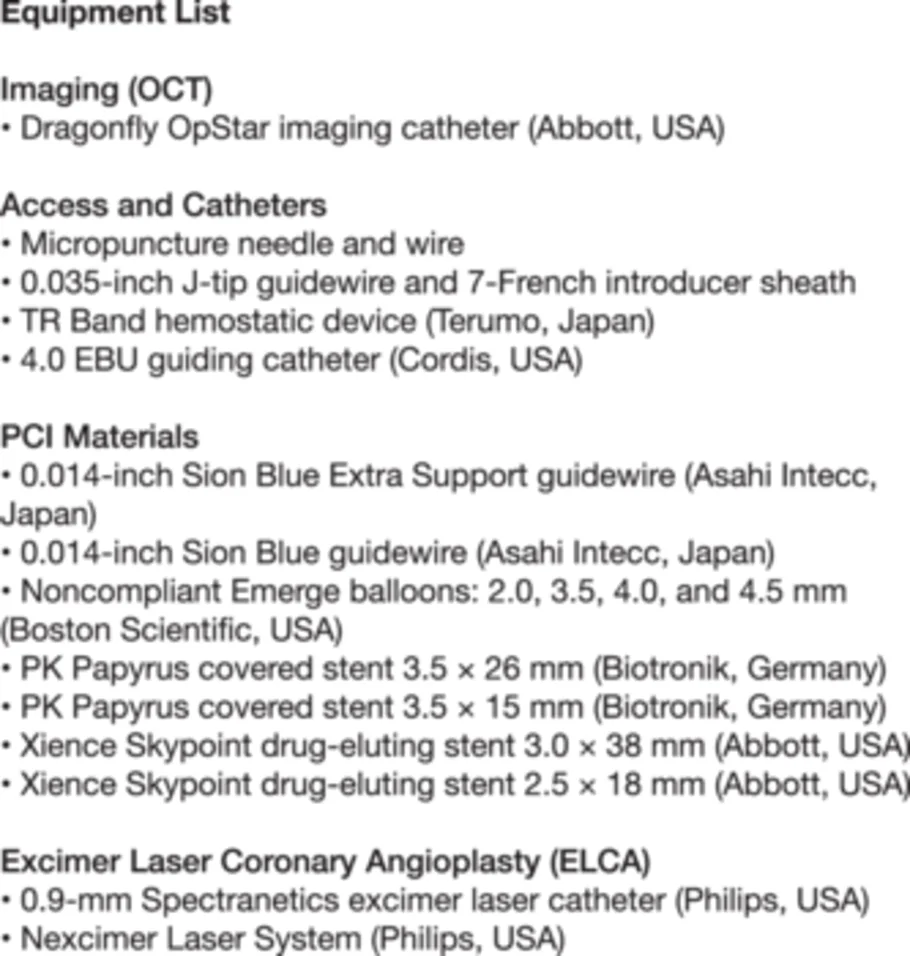
Equipment used for OCT imaging and PCI, including ELCA, Excimer laser coronary angioplasty; OCT, optical coherence tomography; PCI, percutaneous coronary intervention.

**Figure 4:**
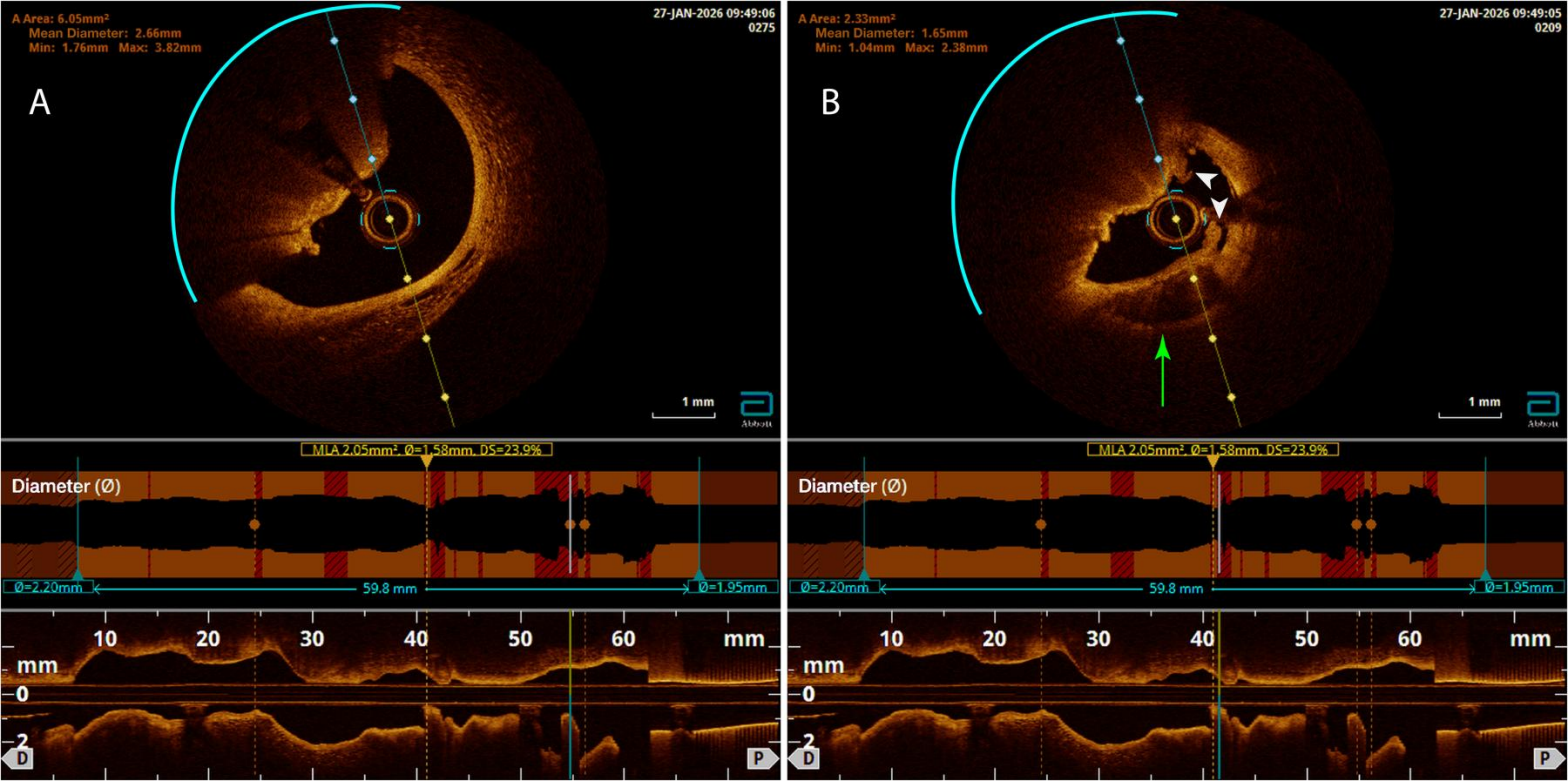
OCT assessment before PCI. (A) Proximal stenosis showing an eruptive calcified nodule with an irregular surface and posterior shadowing (upper left quadrant, light blue line). (B) Distal stenosis showing an eruptive calcified nodule with posterior shadowing (upper left quadrant, light blue line), additional calcium accumulation within the intima–media layer (lower right quadrant, green arrow), and overlying thrombus (white arrowheads). OCT, optical coherence tomography; PCI, percutaneous coronary intervention. [Color figure can be viewed at wileyonlinelibrary.com]

**Figure 5:**
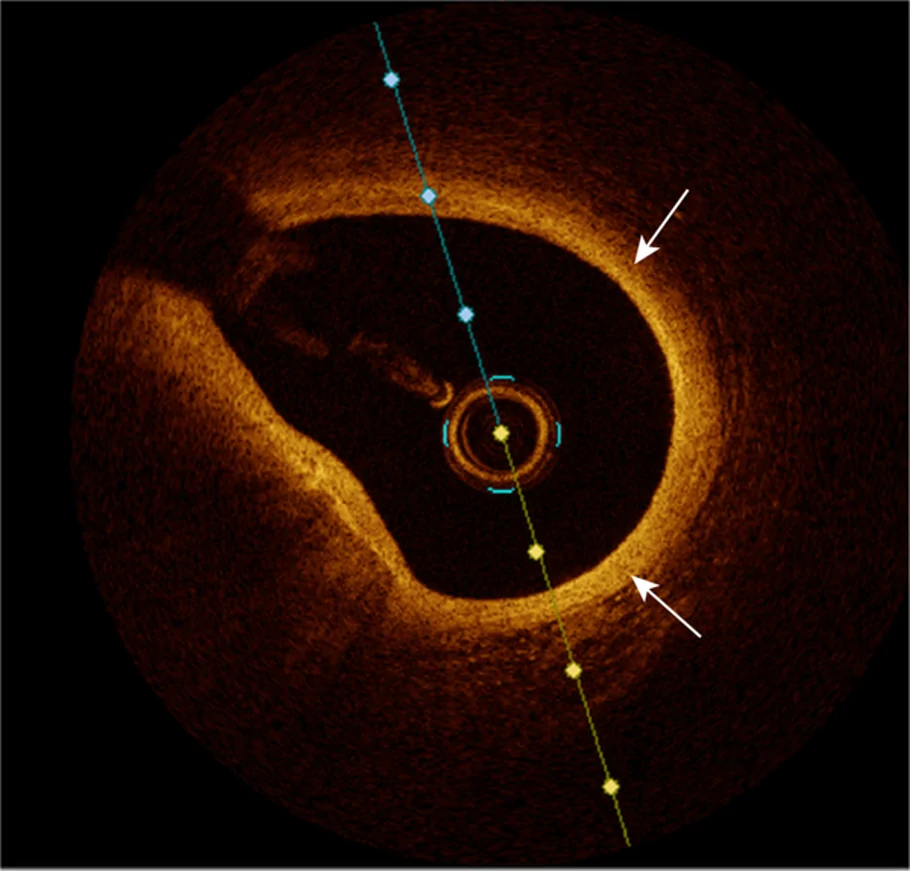
OCT assessment before PCI in a non‐atherosclerotic segment. It is visible intima‐media thickness together with medial thinning (white arrows), finding consistent with radiation‐induced injury. OCT, optical coherence tomography; PCI, percutaneous coronary intervention. [Color figure can be viewed at wileyonlinelibrary.com]

Control angiography showed a large type II coronary perforation with myocardial blush in the proximal LAD and a smaller type II perforation in the mid‐distal LAD (Figure 6 and Video S2). Management included prolonged balloon inflation and implantation of a covered stent (Papyrus 3.5 × 25 mm) in the ostial‐proximal LAD, post‐dilated to 4.5 mm, followed by a drug‐eluting stent (Xience Skypoint 3.0 × 38 mm) in the mid‐LAD. Repeat angiography (Figure 7) demonstrated persistent myocardial blush, whereas OCT showed vessel disruption extending to the external elastic lamina at the distal edge of the first covered stent (Figure 8A) and in the mid‐distal LAD (Figure 8B), along with an intramural hematoma. Control OCT showed no residual eruptive calcified nodules. A second covered stent (Papyrus 3.5 × 15 mm) was implanted with overlap and post‐dilated to 4.5 mm. An additional drug‐eluting stent (Xience Skypoint 2.5 × 18 mm) was placed in the mid‐distal LAD for residual disease and a small dissection (Figure 9). The final angiographic result showed good patency of the implanted stents with preserved flow in the septal and diagonal branches, along with small residual areas of perforation. Further covered stent implantation was deferred to avoid compromising important side branches.

**Figure 6:**
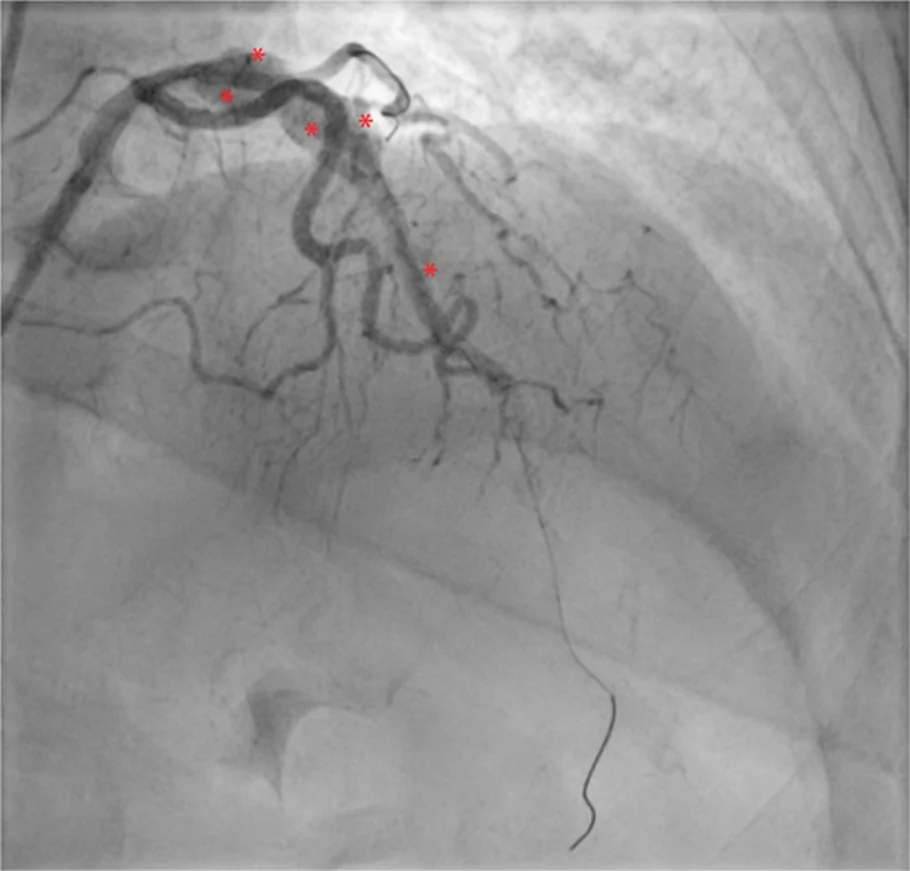
Coronary angiography after ELCA showing multiple coronary perforations with myocardial blush (red asterisks) along the treated proximal and mid segments of the left anterior descending artery. ELCA, Excimer laser coronary angioplasty. [Color figure can be viewed at wileyonlinelibrary.com]

**Figure 7:**
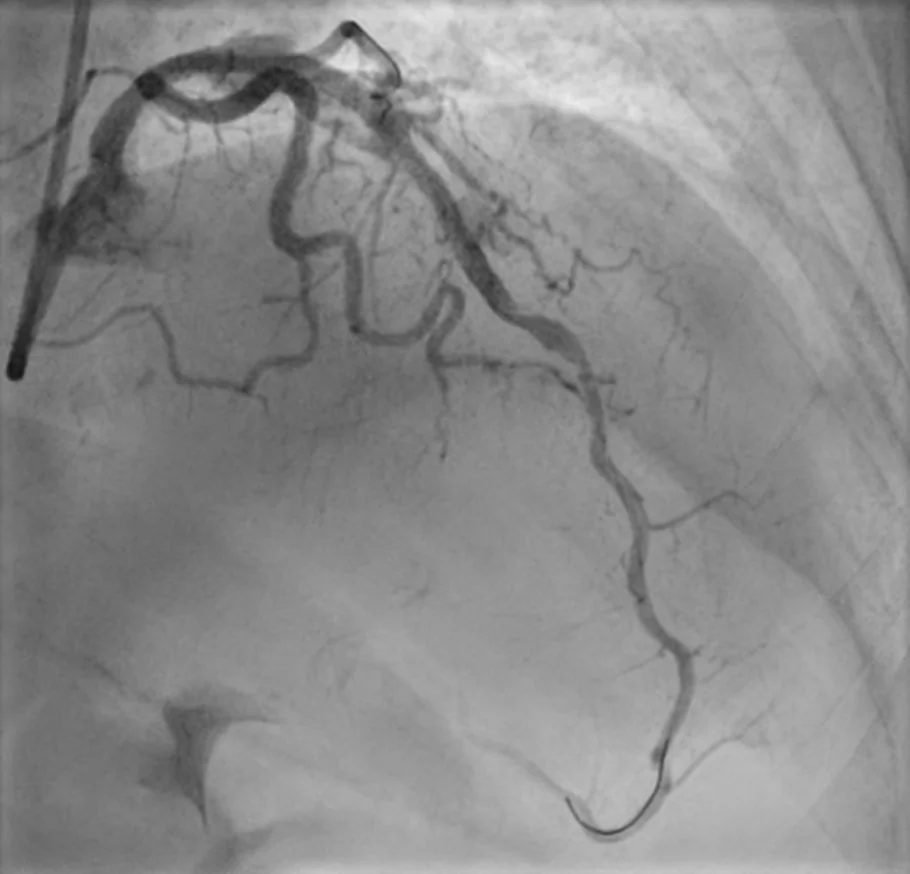
Coronary angiography after implantation of a covered stent in the proximal left anterior descending artery and a drug‐eluting stent in the mid segment, showing persistent myocardial blush.

**Figure 8:**
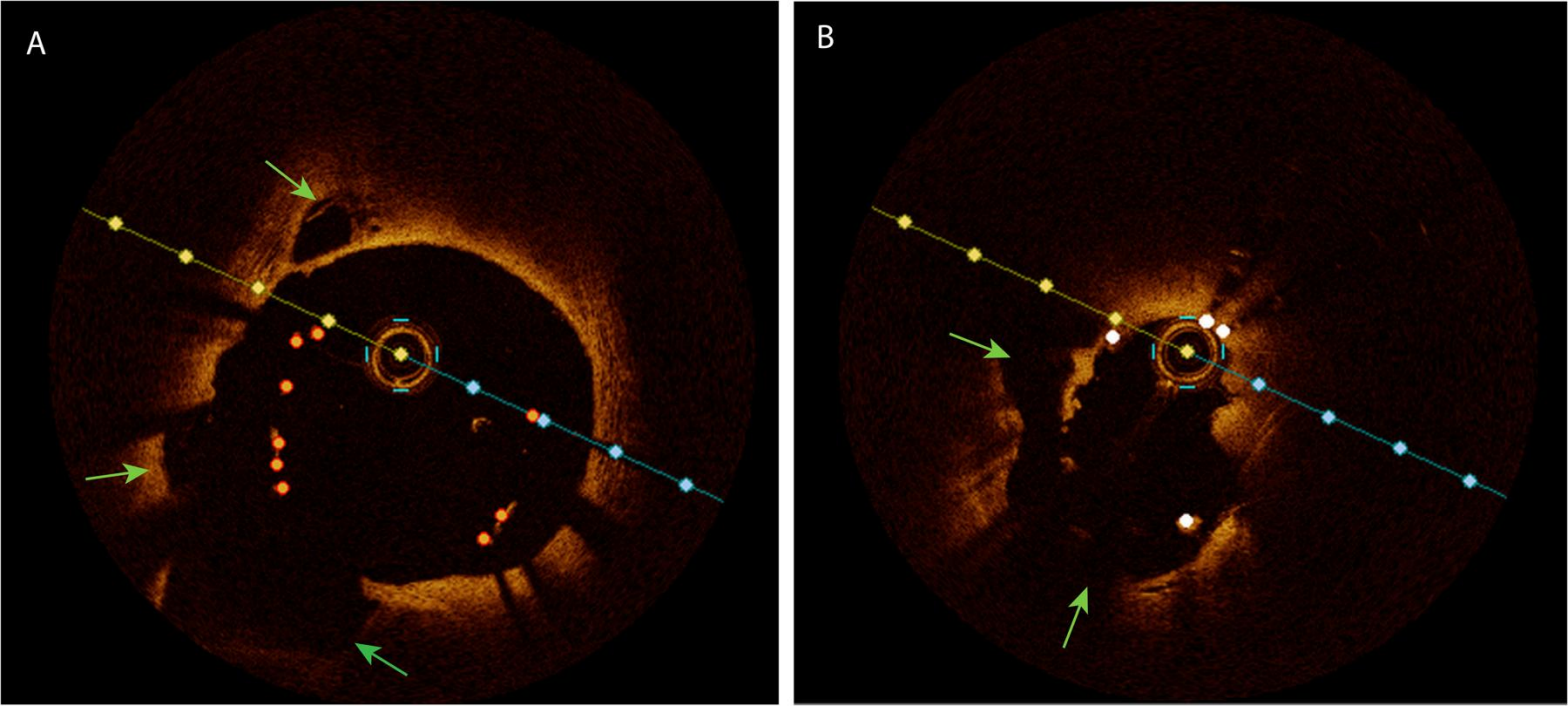
OCT during PCI. (A) Vessel disruption extending to the external elastic lamina at the proximal edge of the drug‐eluting stent (green arrows). (B) Vessel disruption extending to the external elastic lamina at the distal edge of the drug‐eluting stent (green arrows). OCT, optical coherence tomography; PCI, percutaneous coronary intervention. [Color figure can be viewed at wileyonlinelibrary.com]

**Figure 9:**
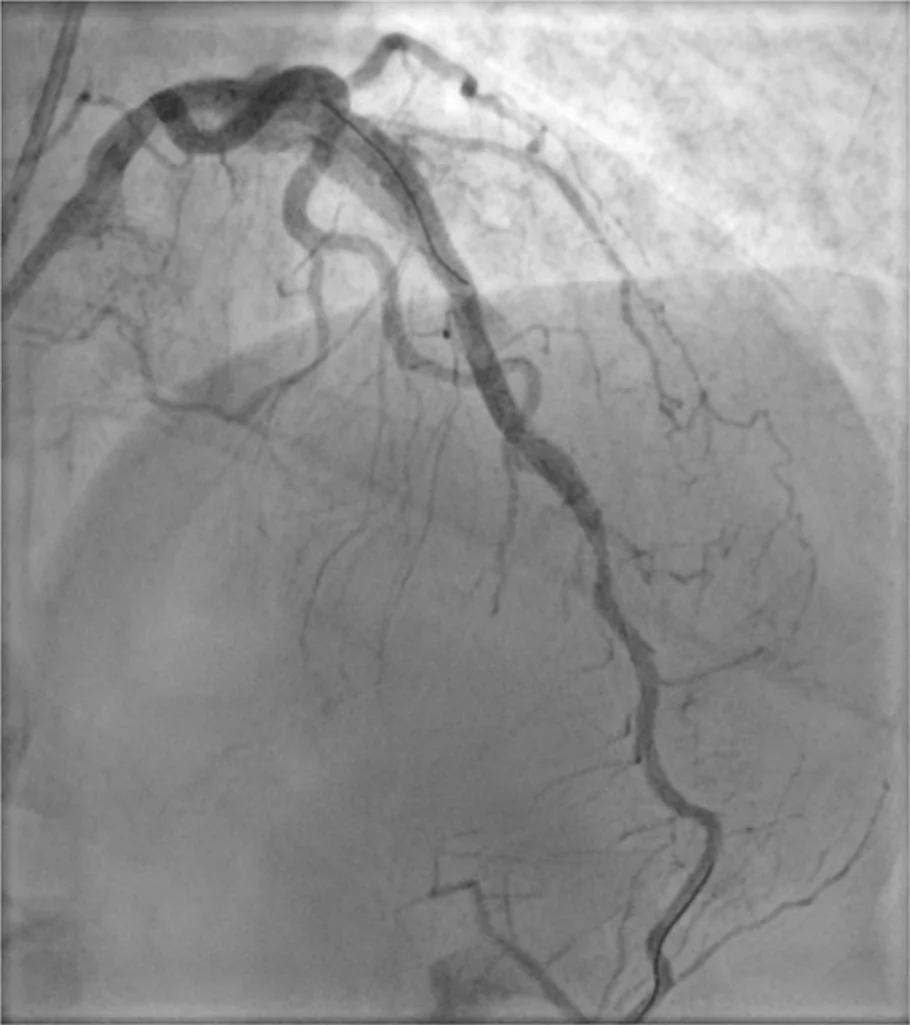
Final coronary angiography after implantation of an additional covered stent in the proximal left anterior descending artery and a drug‐eluting stent in the mid‐distal segment.

The patient remained asymptomatic and hemodynamically stable and was returned to the CICU. The following day, high‐sensitivity troponin I peaked at 23,041 ng/L, with subsequent decline. Medical therapy was optimized, and she was transferred to the cardiology ward. Follow‐up angiography (February 6, 2026) confirmed good stent patency, with more circumscribed areas of residual myocardial blush (Figure 10).

**Figure 10:**
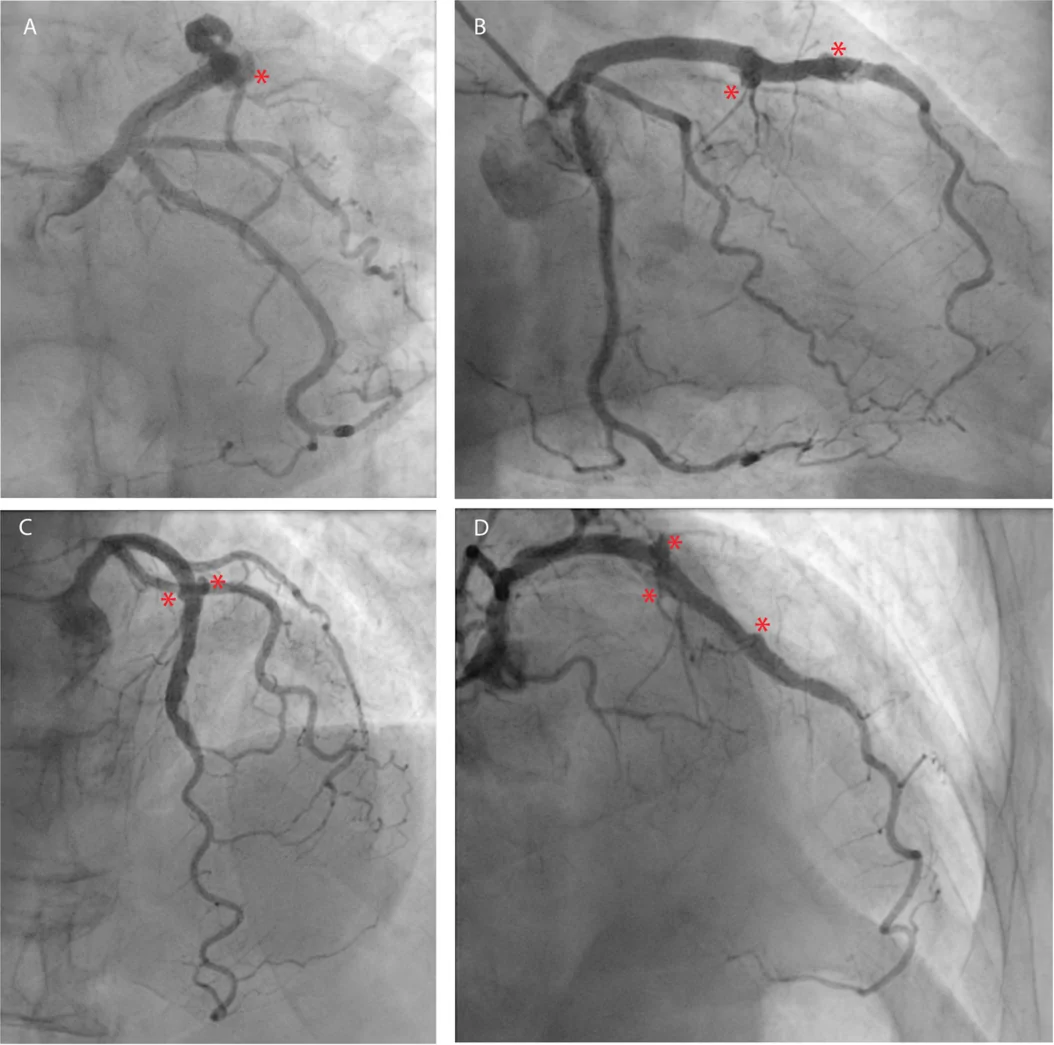
Ten‐day follow‐up coronary angiography showing good patency of the stents implanted in the left anterior descending artery and more circumscribed residual areas of perforation (red asterisks). (A, B) Caudal projections. (C, D) Cranial projections. [Color figure can be viewed at wileyonlinelibrary.com]

## 6 Outcome and Follow‐Up

The patient remained hemodynamically stable throughout hospitalization. Final echocardiography showed persistent LV systolic dysfunction (LVEF 35%) without significant pericardial effusion. She was discharged on February 7, 2026, with continuation of baseline medical therapy, dual antiplatelet therapy, and bisoprolol. In addition, sacubitril/valsartan 24/26 mg twice daily, canrenone 25 mg once daily, and empagliflozin 10 mg once daily were newly initiated.

At 2‐month follow‐up, she was asymptomatic (New York Heart Association class I) with improved LVEF (43%). The lipid profile met secondary prevention targets (LDL cholesterol 46 mg/dL). Sacubitril/valsartan was up‐titrated to 49/51 mg twice daily in the setting of good tolerability and preserved renal function.

## 7 Discussion

This case expands the spectrum of potential mechanisms underlying coronary perforation during ELCA by suggesting a possible contribution of radiation‐related vessel wall remodeling.

Management of heavily calcified coronary lesions remains challenging and is associated with impaired device delivery and suboptimal stent expansion, both linked to worse clinical outcomes [1]. In contemporary practice and according to current expert consensus, calcium modification is recommended before stent implantation, with available options including rotational atherectomy, orbital atherectomy, and intravascular lithotripsy, selected based on lesion characteristics and operator experience rather than by a universally preferred approach [2].

In our case, the lesion was characterized by multiple calcified nodules with superimposed thrombus, making device selection particularly challenging. ELCA acts through photochemical, photothermal, and photomechanical mechanisms, enabling modification of both the calcific and thrombotic components [3]. Laser ablation generates microscopic particulate debris, which may limit distal embolization and the risk of slow‐flow/no‐reflow [4, 5, 6], an important consideration in a patient with severely reduced left ventricular ejection fraction. Furthermore, the unfavorable guidewire bias made rotational atherectomy less attractive, supporting the choice of ELCA in this complex anatomy.

In this patient, although multiple traditional cardiovascular risk factors were present, prior radiotherapy may have contributed to vessel wall remodeling that extends beyond the atherosclerotic process. Radiation‐induced vascular injury begins within days after irradiation with endothelial injury, inflammation, oxidative stress, and apoptosis, whereas extracellular matrix deposition and progressive fibrosis evolve over subsequent months, involving all layers of the arterial wall [7, 8, 9]. These changes reduce vascular elasticity and mechanical integrity and may promote accelerated atherosclerosis, although radiation‐associated coronary artery disease typically becomes clinically apparent only years after exposure [10]. Histopathological studies have shown that irradiated arteries exhibit increased intima‐media thickness together with medial thinning and adventitial fibrosis, suggesting a pattern of vessel wall remodeling beyond conventional atherosclerosis [9], as also observed on OCT imaging in our case.

In this context, laser energy delivery may have represented an additional contributing factor. Although several measures were adopted to minimize the risk of vessel injury—including the use of the smallest available laser catheter (0.9 mm), slow laser advancement and retraction, continuous saline flushing without contrast injection, and treatment of a non‐tortuous vessel [3, 4]—the prolonged delivery of high‐energy laser pulses and the complexity of the heavily calcified lesions may also have contributed to the coronary perforations. At the same time, vessel disruption extending to the external elastic lamina in multiple LAD segments raises the possibility of an underlying diffuse susceptibility of the vessel wall.

Therefore, this case suggests that coronary perforation during ELCA may result from the interplay between lesion complexity, procedural factors, and patient‐specific vessel characteristics, including prior thoracic radiotherapy. In the absence of specific recommendations for irradiated coronary vessels, careful device selection, individualized energy delivery, and stepwise lesion preparation appear warranted.

## 8 Conclusions

Although ELCA is an effective plaque‐modification technique for selected complex coronary lesions, this case suggests that prior thoracic radiotherapy may influence vessel wall susceptibility and should be considered when planning complex plaque‐modification procedures. Further studies are warranted to better define the impact of radiation‐related vessel remodeling on procedural risk and device selection in this challenging population.

## Conflicts of Interest

The authors declare no conflicts of interest.

## Supporting information


**Video S1:** Two runs of antegrade ELCA in the proximal and mid segments of the left anterior descending artery, with smooth lesion crossing. Laser activation was delivered in 10‐second bursts followed by 5‐second pauses (0.9‐mm catheter) to reduce the risk of complications. *ELCA: excimer laser coronary angioplasty*.

**Video S2:** Post‐ELCA coronary angiography demonstrating multiple coronary perforations with associated myocardial blush along the treated proximal and mid segments of the left anterior descending artery. *ELCA: excimer laser coronary angioplasty*.

## Data Availability

The data that support the findings of this study are available on request from the corresponding author. The data are not publicly available due to privacy or ethical restrictions.
